# Phase Stability and Mechanical Properties of Al_8_Fe_4_RE via First-Principle Calculations

**DOI:** 10.3390/ma12050701

**Published:** 2019-02-27

**Authors:** Rongcheng Wang, Xiaoma Tao, Hongmei Chen, Yifang Ouyang

**Affiliations:** 1 School of Materials Science and Engineering, South China University of Technology, Guangzhou 510640, China; rong_phy@163.com; 2 Guangxi Key Laboratory of Processing for Non-ferrous Metallic and Featured Materials, School of Physical Science and Technology, Guangxi University, Nanning 530004, China; taoxiaoma@gxu.edu.cn (X.T.); chenhm@gxu.edu.cn (H.C.)

**Keywords:** first-principle calculation, Al_8_Fe_4_RE, elastic properties, lattice dynamic

## Abstract

We report on the phase stability, elastic, electronic, and lattice dynamic properties of 17 Al_8_Fe_4_RE (RE = Sc, Y, La–Lu) intermetallic compounds (IMCs) using first-principle calculations. The calculated lattice constants coincided with the experimental results. The calculated enthalpy formation indicated that all the 17 IMCs are stable. The elastic constants and various moduli indicated that Al_8_Fe_4_RE can be used as a strengthening phase due to its high Young’s modulus and shear modulus. The 3D surfaces of Young’s modulus for Al_8_Fe_4_RE showed anisotropic behavior, and the values of hardness for the IMCs were high (about 14 GPa). The phonon spectra showed that only Al_8_Fe_4_Y had a soft mode, which means the other IMCs are all dynamically stable.

## 1. Introduction

Due to their low density, low thermal conductivity, relative high strength, and low material cost, Al–Fe-based alloys have been studied extensively over the last few decades [1,2,3,4]. Al–Fe-based alloys are promising, high-temperature structural materials; however, their limited ductility at room temperature and the reduction in strength above 600 °C obstruct their application as high-temperature structural materials. Nevertheless, some recent investigations have shown that the mechanical properties can be effectively improved by controlling the microstructure, composition, and alloying elements [5,6,7,8].

As we known, rare-earth (RE) elements are special modifiers that are commonly used in Al-based and Fe-based alloys. Thus, the addition of RE elements in Al–Fe-based alloys may affect the microstructure and improve the mechanical properties of these alloys. When RE elements are added, Al–Fe–RE intermetallic compounds (IMCs) form, which affects the phase relationship and microstructure of Al–Fe-based alloys. The mechanical properties of Al–Fe-based alloys are consequently improved due to the changes in composition and microstructure. In previous works, Al–Fe–RE (RE = Y, Ce, Nd, Gd, Er) ternary phase diagrams have been experimentally investigated [9,10,11,12,13], and the ternary IMCs have been determined. The Al_8_Fe_4_RE IMCs are observed at the Al-rich corner, and they have a tetragonal crystal structure. The 17 Al_8_Fe_4_RE (RE = Sc, Y, La, Ce, Nd, Eu–Er, and Lu) IMCs have also been previously determined in experiments [14,15,16,17,18,19,20,21,22,23,24,25]. Using the empirical electron theory (EET), Al_8_Fe_4_Ce was found to be favorable for the stability of the Al-based alloy as a strengthening phase [15]. The magnetic properties of Al_8_Fe_4_RE have also been investigated [16,17,18,19,20,21], and the electronic conductivity [22] and the negative magnetoresistivity [23] of Al_8_Fe_4_RE have also been studied. Using the lattice inversion method, the lattice constants and lattice vibration spectra of Al_8_Fe_4_RE (RE = Sc, Ce, Nd, Sm) have been reported [24,25]. As a potential strengthening phase and as magnetic materials, the structural stability and electronic and elastic properties of Al_8_Fe_4_RE are very important for material design and for further development. However, few studies have focused on the electronic and elastic properties of Al_8_Fe_4_RE IMCs. Thus, the aim of this work was to study the physical properties of 17 Al_8_Fe_4_RE (RE = Sc, Y, and La–Lu) IMCs using first-principle (FP) calculations.

## 2. Computational Details

The FP calculations were performed with the VASP code [26,27] using the projector augmented wave (PAW) method [28,29] and the generalized gradient approximation (GGA) [30]. The GGA-PBE (Generalized Gradient Approximation-Perdew–Burke–Ernzerhof) potentials of Al, Fe, Sc, Y_sv, La_s, Yb_2, and RE_3 (others) were used in this work. The FP calculations were performed with cutoff energy of 500 eV, Monkhorst–Pack K-point meshes [31], and a 0.05 eV smearing parameter with the Methfessel–Paxton technique [32]. 

The formation enthalpy and cohesive energy of the Al_8_Fe_4_RE alloys can be estimated from the following equations:

(1) $$\Delta H\left(Al_{8}Fe_{4}RE\right)=E\left(Al_{8}Fe_{4}RE\right)-8E\left(Al\right)-4E\left(Fe\right)-E\left(RE\right)$$

(2) $$E_{c}\left(Al_{8}Fe_{4}RE\right)=E\left(Al_{8}Fe_{4}RE\right)-8E_{single}\left(Al\right)-4E_{single}\left(Fe\right)-E_{single}\left(RE\right)$$

where *E*(*Al*_8_*Fe*_4_*RE*), *E*(*Al*), *E*(*Fe*), and *E*(*RE*) are the equilibrium first-principles-calculated total energies of the Al_8_Fe_4_RE IMCs, Al, Fe, and rare earth element, respectively. In the calculation, the Al, Ce, and Yb have the face-centered cubic (FCC) structure, Fe and Eu have the body-centered cubic (BCC) structure, and the others have the hexagonal close packed (HCP) structure. The *E_single_*(*Al*), *E_single_*(*Fe*), and *E_single_*(*RE*) are the total energies of the isolated atoms.

For a tetragonal structure, there are six independent single-crystal elastic constants: *C*_11_, *C*_12_, *C*_33_, *C*_13_, *C*_44_, and *C*_66_. The calculated details can be found in [33] and are not recalled here. The effective elastic moduli can be estimated with Voigt [34], Reuss [35], and Hill [36] methods. Usually, the Voigt–Reuss–Hill (VRH) value is used as an effective data [37]. 

## 3. Results and Discussion

### 3.1. Phase Stability

The lattice constants, formation enthalpies, cohesive energies of 17 Al_8_Fe_4_RE IMCs were calculated, and the obtained results are listed in Table 1 with experimental [14] and theoretical data [38]. It can be seen from Table 1 that the calculated lattice constants of Al_8_Fe_4_RE IMCs were all in coincident with the experimental data [14], and the lattice constants slightly reduced with the increase in atomic number, which is known as the “lanthanide contraction”. This phenomenon occurs in RE pure elements and RE-bearing IMCs [39,40,41]. The formation enthalpies (Δ*H*) and cohesive energies (*E*_c_) of Al_8_Fe_4_RE IMCs were all negative, showing that all the Al_8_Fe_4_RE IMCs are stable. For Al_8_Fe_4_Gd, the formation energy of CALPHAD is −0.6114 eV/atom [38], and the calculated result was −0.4254 eV/atom. As we known, the CALPHAD data is estimated from some experimental phase and thermodynamic data, which is the reason for the difference in the two results. However, some further experiments are needed to validate the calculated ΔH and *E*_c_ of Al_8_Fe_4_RE. The magnetic moments of Al_8_Fe_4_RE were also obtained. The magnetic moments changed from 1.4 to 1.6 *μ*_B_ per Fe atom. Here, it should be noted that the RE_3 with *f*-electrons were kept frozen in core used in the present work.

### 3.2. Mechanical Properties

In order to shed some light on the mechanical properties of Al_8_Fe_4_RE IMCs, the elastic constants (*C*_ij_) of Al_8_Fe_4_RE IMCs were calculated, and the results are listed in Table 2.

Obviously, the present elastic constants *C*_ij_ of the 17 Al_8_Fe_4_RE IMCs met the requirement of stability conditions with *C*_11_ > 0, *C*_33_ > 0, *C*_44_ > 0, *C*_66_ > 0, (*C*_11_ − *C*_12_) > 0, (*C*_11_ + *C*_33_ − 2*C*_13_) > 0, and [2(*C*_11_ + *C*_12_) + *C*_33_ + 4*C*_13_] > 0. For Al_8_Fe_4_RE (RE = Sc, La, Ce, Pr, Yb), *C*_11_ < *C*_33_ indicated that the bonding strength along the [100] and [010] directions was softer than that along the [001] direction. However, for the others, *C*11 > *C*33, the opposite tendency occurred. *C*44 < *C*66 meant the [100](001) shear was easier than the [100](010) shear for the 17 Al_8_Fe_4_RE IMCs. 

**Figure 1:**
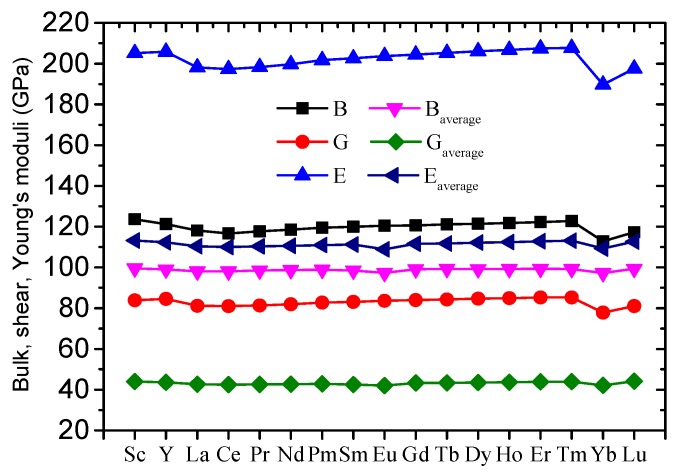
The calculated bulk, shear, and Young’s modulus of Al_8_Fe_4_RE IMCs.

The bulk modulus (B), shear modulus (G), Young’s modulus (E), and Poisson’s ratio (σ) of the 17 Al_8_Fe_4_RE IMCs were estimated, and the results are listed in Table 3. The bulk moduli (B) of the 17 Al_8_Fe_4_RE IMCs were larger than that of Al (72 GPa) [42], and the shear moduli (G) and Young’s moduli (E) of the 17 Al_8_Fe_4_RE IMCs were three times that of Al (27 GPa and 71 GPa) [42]. In order to compare them clearly, the arithmetic average values of pure Al, Fe, and RE with the weight of composition were calculated for Al_8_Fe_4_RE IMCs, and the results are shown in Figure 1. Obviously, the presently calculated bulk moduli (B) were 1.2 times than that of the arithmetic average values, and the presently calculated G and E were close to two times the arithmetic average values. This indicates that the Al_8_Fe_4_RE IMCs may be used as a strengthening phase. 

**Figure 2:**
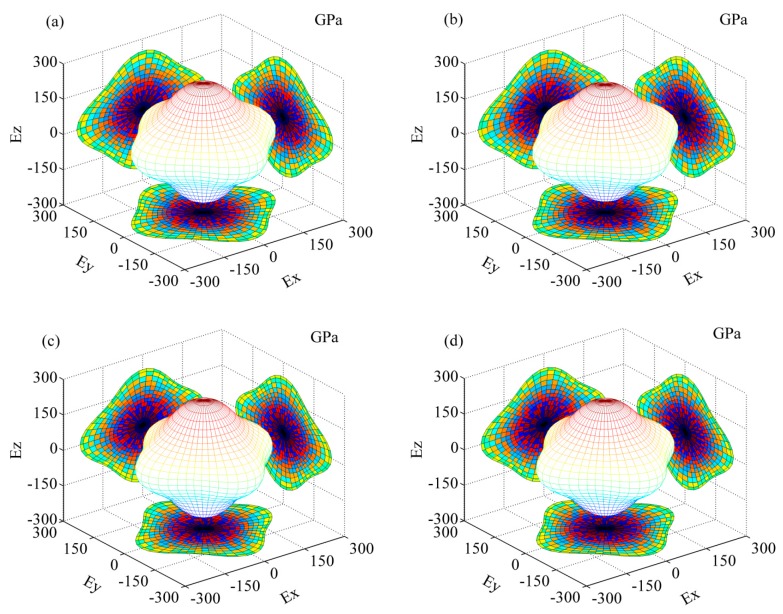

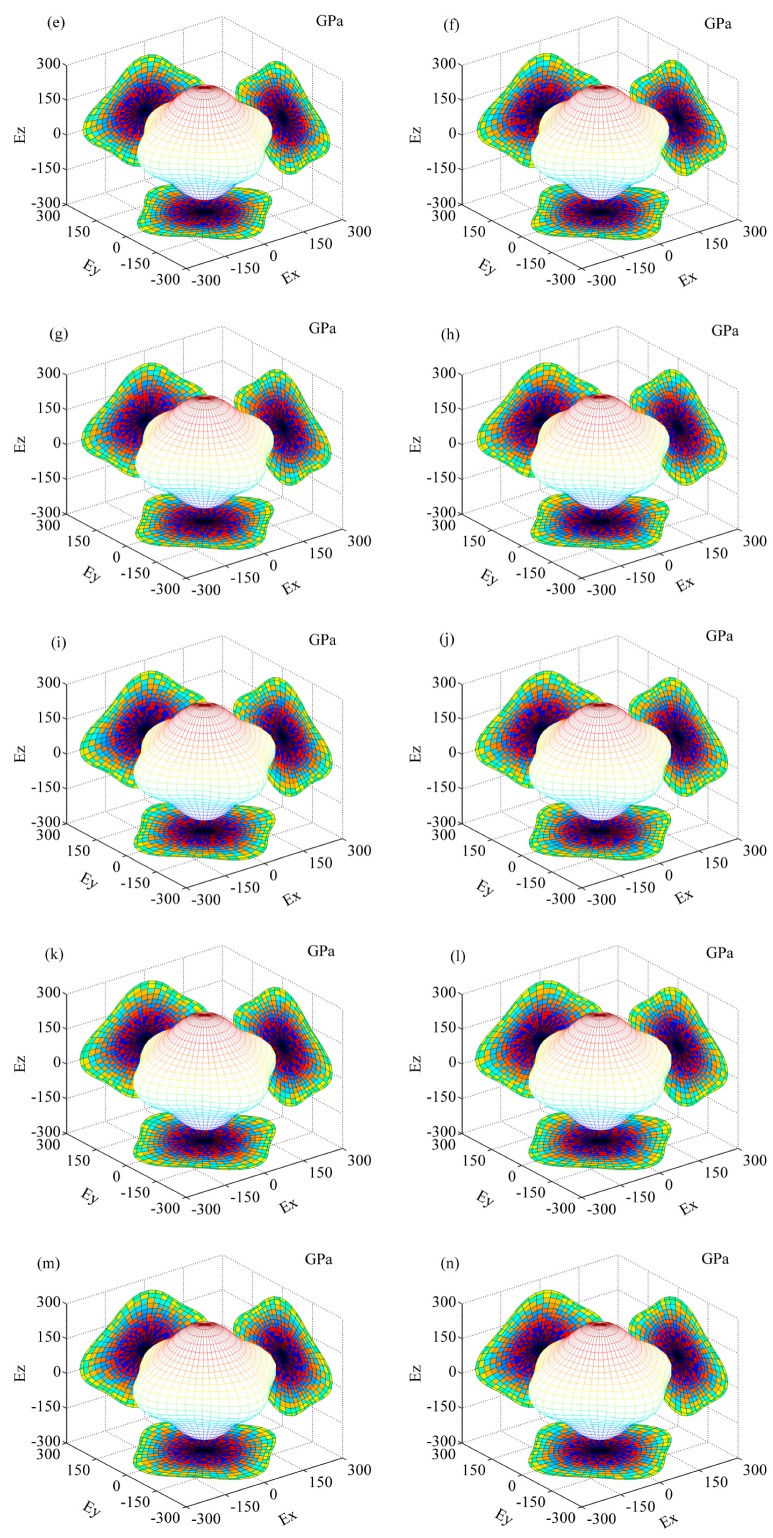

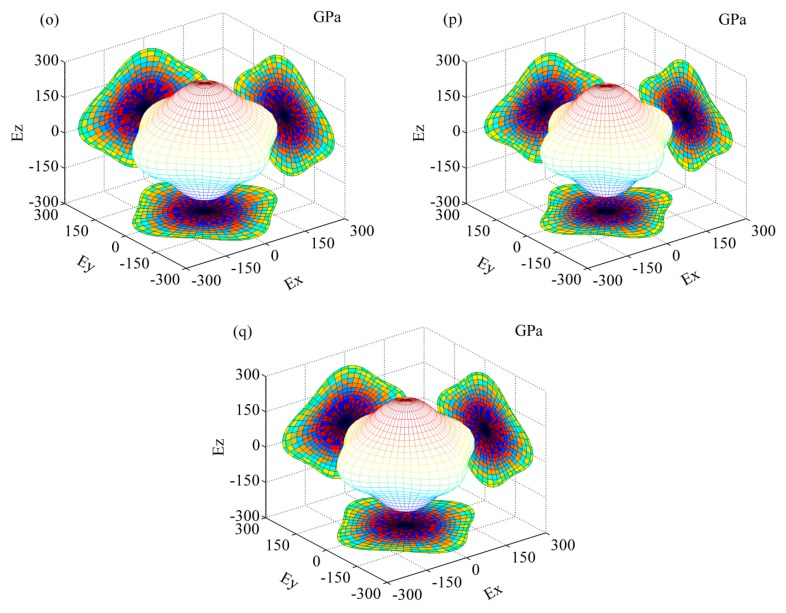
The 3D curved surface of the Young’s modulus of Al_8_Fe_4_RE IMCs. (**a**) Al_8_Fe_4_Sc; (**b**) Al_8_Fe_4_Y; (**c**) Al_8_Fe_4_La; (**d**) Al_8_Fe_4_Ce; (**e**) Al_8_Fe_4_Pr; (**f**) Al_8_Fe_4_Nd; (**g**) Al_8_Fe_4_Pm; (**h**) Al_8_Fe_4_Sm; (**i**) Al_8_Fe_4_Eu; (**j**) Al_8_Fe_4_Gd; (**k**) Al_8_Fe_4_Tb; (**l**) Al_8_Fe_4_Dy; (**m**) Al_8_Fe_4_Ho; (**n**) Al_8_Fe_4_Er; (**o**) Al_8_Fe_4_Tm; (**p**) Al_8_Fe_4_Yb; (**q**) Al_8_Fe_4_Lu.

In order to illustrate the elastic anisotropy of Al_8_Fe_4_RE IMCs, the surfaces of Young’s modulus for Al_8_Fe_4_RE IMCs are shown in Figure 2. The three-dimensional surface exhibited a spherical shape for an isotropic crystal. As can be seen in Figure 2, the isosurfaces of Young’s modulus exhibited remarkable anisotropic behavior for all the Al_8_Fe_4_RE IMCs. In the light of the Pugh criterion [43], the B/G ratios for the 17 Al_8_Fe_4_RE IMCs were all smaller than 1.75, which reveals that the IMCs are prone to brittleness. A theoretical model [44] of linking Vickers hardness and moduli is via *Hv* = 2 × (*k*^−2^G)^0.585^ − 3, where *Hv* is Vickers hardness and *k* is the ratio B/G. The calculated Vickers hardness of the 17 Al_8_Fe_4_RE IMCs were all about 14 GPa.

### 3.3. Electronic Properties

**Figure 3:**
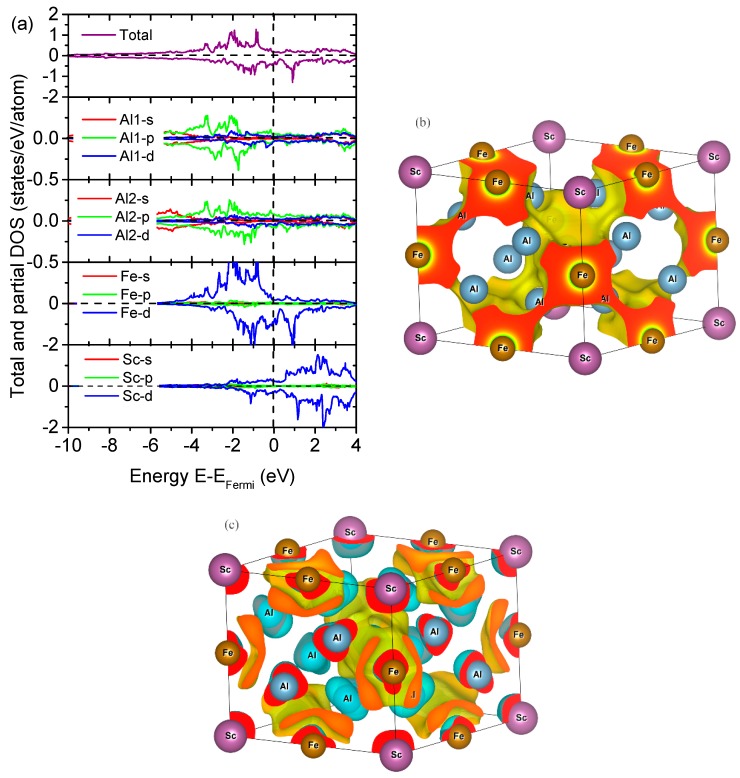
(**a**) The total and partial density of states, (**b**) electron localization function, and (**c**) bonding charge density of Al_8_Fe_4_Sc.

The density of states (DOS), electron localization function (ELF), and bonding charge density (BCD) for Al_8_Fe_4_Sc are plotted in Figure 3 as an example. It can be seen from Figure 3 that Al_8_Fe_4_Sc showed metallic behavior, and the DOS at the Fermi level was mainly dominated by the Fe-3d state and Sc-3d states, evidencing the hybridization at the Fermi level. The ELF and BCD showed a depletion of the electronic density (ED) at the Al and Sc lattice sites, along with an increment of the ED at the Fe sites. This feature is consistent with the DOS plots in Figure 3a, demonstrating the hybridization of Fe-3d and Sc-3d. For the other Al_8_Fe_4_RE IMCs, their electronic structures were all similar to Al_8_Fe_4_Sc, so they are not shown here (see Supplementary data).

### 3.4. Lattice Dynamical Properties

**Figure 4:**
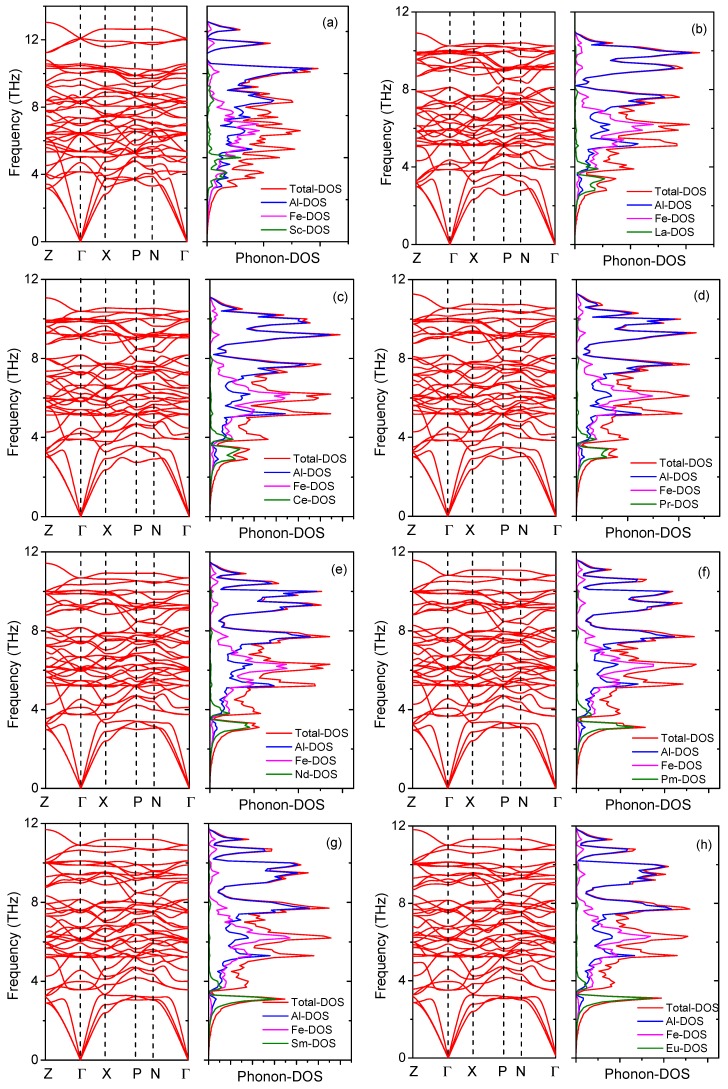

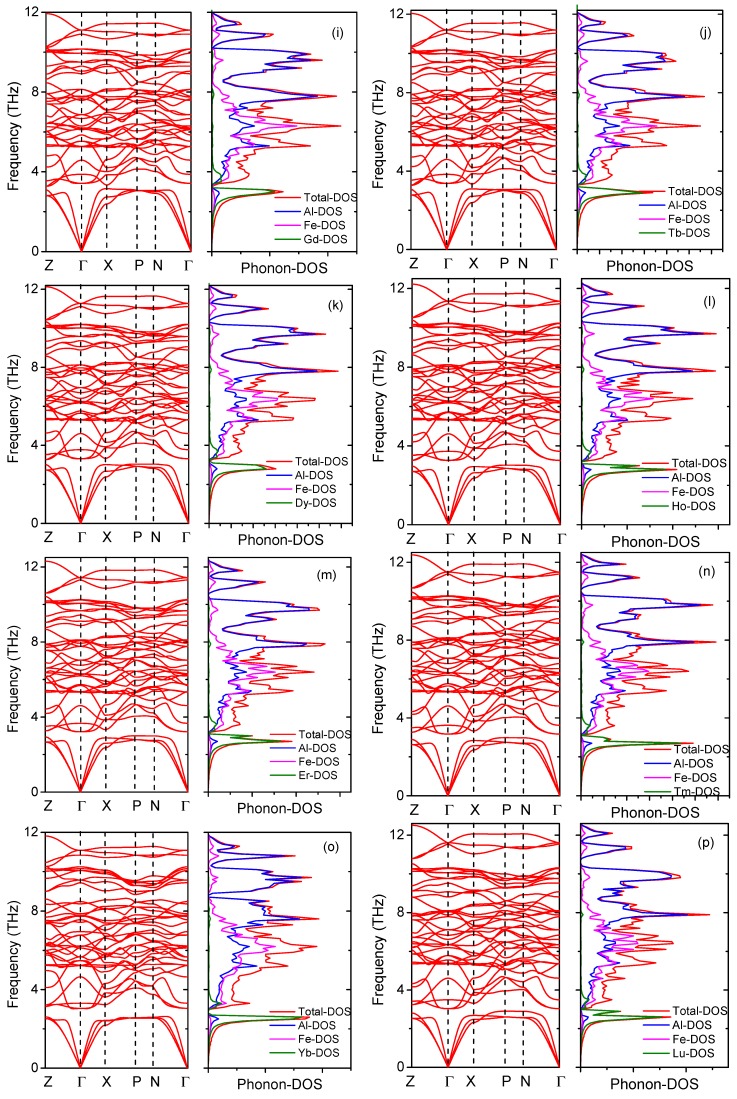
Phonon dispersion spectrum and phonon density of state for Al_8_Fe_4_RE(RE = Sc, La–Lu) IMCs. (**a**) Al_8_Fe_4_Sc; (**b**) Al_8_Fe_4_La; (**c**) Al_8_Fe_4_Ce; (**d**) Al_8_Fe_4_Pr; (**e**) Al_8_Fe_4_Nd; (**f**) Al_8_Fe_4_Pm; (**g**) Al_8_Fe_4_Sm; (**h**) Al_8_Fe_4_Eu; (**i**) Al_8_Fe_4_Gd; (**j**) Al_8_Fe_4_Tb; (**k**) Al_8_Fe_4_Dy; (**l**) Al_8_Fe_4_Ho; (**m**) Al_8_Fe_4_Er; (**n**) Al_8_Fe_4_Tm; (**o**) Al_8_Fe_4_Yb; (**p**) Al_8_Fe_4_Lu.

**Figure 5:**
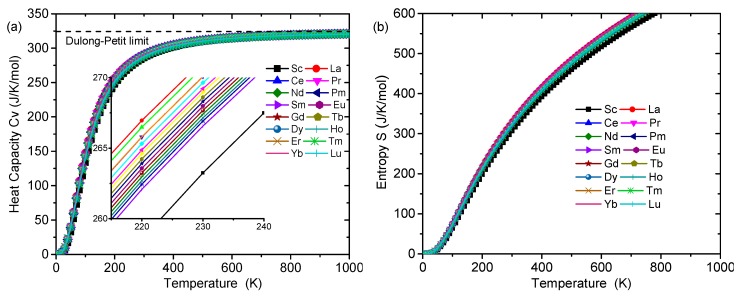
The (**a**) heat capacity and (**b**) entropy of Al_8_Fe_4_RE (RE = Sc, La–Lu) IMCs.

In order to check the dynamic stability, the phonon dispersion (PD) curves of Al_8_Fe_4_RE IMCs were calculated by combing VASP and PHONOPY codes [45]. For the PD calculation, we used 2 × 2 × 2 supercell containing 104 atoms for Al_8_Fe_4_RE and 5 × 5 × 5 k-point mesh. The calculated PD curves along Z-Γ-X-P-N-Γ directions and the phonon density of states (PDOS) are plotted in Figure 4. Among the 17 Al_8_Fe_4_RE IMCs, only Al_8_Fe_4_Y had the imaginary frequency, indicating that Al_8_Fe_4_Y is dynamically unstable. For the others, the calculated PD curves did not have any soft mode, confirming the dynamic stability of Al_8_Fe_4_RE (RE, La–Lu) IMCs. The heat capacity *C*_v_ and entropy *S* of Al_8_Fe_4_RE IMCs are shown in Figure 5. The calculated *C*_v_ exhibited the expected *T*^3^ power law in the low temperature, and *C*_v_ reached a classic limit of 324.246 J·(K·mol)^−1^ at high temperature, which is consistent with the classic law of Dulong–Petit. However, no experimental data of *C*_v_ of Al_8_Fe_4_RE could be found in the literatures. The present calculations should be a prediction, and further experiments are needed in the future.

## 4. Summary

Using the first-principle calculations, the phase stability, elastic constants, various moduli, hardness, electronic, and lattice dynamical properties of Al_8_Fe_4_RE IMCs were investigated. The calculated lattice constants were all consistent with the experimental data. The formation enthalpies were all negative, meaning all the IMCs are stable from a thermodynamic point view. The calculated Young’s and shear modulus were three times as large as that of Al and two times as large as that of arithmetic average values. The values of hardness of Al_8_Fe_4_RE IMCs were all about 14 GPa. All of abovementioned mechanical properties indicate that Al_8_Fe_4_RE IMCs may be a good strengthening phase for Al–Fe-based alloys. The calculated PD of Al_8_Fe_4_Y had a soft mode, and the others had no soft mode at any vectors, which means that Al_8_Fe_4_Y is dynamically unstable, while the others are all dynamically stable. The results are beneficial for the extensive application of Al_8_Fe_4_RE IMCs.

## Figures and Tables

**Table 1:** Lattice constants, formation enthalpy, cohesive energy, and magnetic moments of Al_8_Fe_4_RE IMCs.

Phases	Lattice Constants		△*H* (eV/atom)	*Ec* (eV/atom)	Magnetic (*μ*_B_/Fe)	Ref.
	*a* (Å)	*c* (Å)				
Al_8_Fe_4_Sc	8.597 8.70	5.001 4.81	−0.4238	−4.5211	1.426	Present [14]
Al_8_Fe_4_Y	8.696 8.750	5.024 5.060	−0.4322	−4.5221	1.500	Present [14]
Al_8_Fe_4_La	8.849 8.900	5.045 5.075	−0.3843	−4.4922	1.602	Present [14]
Al_8_Fe_4_Ce	8.829 8.793	5.046 5.047	−0.3843	−4.4963	1.599	Present [14]
Al_8_Fe_4_Pr	8.802 8.824	5.042 5.054	−0.3955	−4.5076	1.584	Present
Al_8_Fe_4_Nd	8.781 8.804 8.875	5.039 5.054 5.211	−0.4043	−4.5152	1.571	Present [14,24]
Al_8_Fe_4_Pm	8.762	5.035	−0.4122	−4.5173	1.559	Present
Al_8_Fe_4_Sm	8.748 8.770 8.863	5.032 5.053 5.188	−0.4162	−4.5223	1.549	Present [14,24]
Al_8_Fe_4_Eu	8.732 8.784	5.035 5.051	−0.4328	−4.5259	1.536	Present [14]
Al_8_Fe_4_Gd	8.719 8.743	5.028 5.052	−0.4254 −0.6114	−4.5283	1.524	Present [14,38]
Al_8_Fe_4_Tb	8.708 8.740	5.024 5.036	−0.4277	−4.5283	1.511	Present [14]
Al_8_Fe_4_Dy	8.697 8.728	5.022 5.050	−0.4291	−4.5275	1.499	Present [14]
Al_8_Fe_4_Ho	8.688 8.720	5.021 5.038	−0.4298	−4.5262	1.488	Present [14]
Al_8_Fe_4_Er	8.678 8.700	5.018 5.028	−0.4296	−4.5247	1.477	Present [14]
Al_8_Fe_4_Tm	8.669 8.688	5.016 5.037	−0.4288	−4.5224	1.466	Present [14]
Al_8_Fe_4_Yb	8.703 8.691	5.049 5.017	−0.3652	−4.2378	1.559	Present [14]
Al_8_Fe_4_Lu	8.653 8.687	5.012 5.030	−0.4256	−4.5174	1.450	Present [14]

**Table 2:** The calculated elastic constants of Al_8_Fe_4_RE intermetallic compounds (IMCs) (unit in GPa).

Phases	*C*_11_	*C*_12_	*C*_13_	*C*_33_	*C*_44_	*C*_66_
Al_8_Fe_4_Sc	266.15	49.09	53.62	268.07	68.43	76.57
Al_8_Fe_4_Y	264.94	46.05	52.36	259.71	70.02	77.63
Al_8_Fe_4_La	254.17	41.90	52.25	262.29	67.73	70.86
Al_8_Fe_4_Ce	252.33	42.85	51.22	255.08	68.08	71.60
Al_8_Fe_4_Pr	255.29	43.90	51.40	255.34	68.35	71.43
Al_8_Fe_4_Nd	257.70	44.72	51.42	256.22	68.77	71.96
Al_8_Fe_4_Pm	260.29	45.46	51.61	257.71	69.33	73.14
Al_8_Fe_4_Sm	261.44	45.87	51.76	258.24	69.50	73.91
Al_8_Fe_4_Eu	262.97	46.12	51.84	258.83	69.74	74.79
Al_8_Fe_4_Gd	263.60	46.01	51.87	258.92	69.75	76.04
Al_8_Fe_4_Tb	264.58	46.22	52.25	259.89	69.80	76.95
Al_8_Fe_4_Dy	265.29	46.15	52.38	260.28	70.09	77.59
Al_8_Fe_4_Ho	265.91	46.22	52.78	261.12	70.39	78.01
Al_8_Fe_4_Er	266.61	46.29	53.14	261.95	70.74	78.24
Al_8_Fe_4_Tm	267.35	46.46	53.64	262.82	70.90	77.65
Al_8_Fe_4_Yb	247.62	38.03	48.66	249.15	64.34	66.59
Al_8_Fe_4_Lu	254.53	44.55	51.63	250.65	67.35	73.88

**Table 3:** The calculated bulk modulus B, shear modulus G, Young’s modulus E, Poisson’s ratio *v*, B/G ratio, and hardness H of Al_8_Fe_4_RE IMCs.

Phases	B (GPa)	G (GPa)	E (GPa)	*v*	B/G	H
Al_8_Fe_4_Sc	123.66	83.85	205.17	0.224	1.475	13.94
Al_8_Fe_4_Y	121.23	84.56	205.82	0.217	1.434	14.59
Al_8_Fe_4_La	118.10	81.19	198.17	0.220	1.454	13.90
Al_8_Fe_4_Ce	116.68	81.01	197.36	0.218	1.440	14.07
Al_8_Fe_4_Pr	117.69	81.36	198.36	0.219	1.447	14.02
Al_8_Fe_4_Nd	118.52	81.93	199.76	0.219	1.447	14.09
Al_8_Fe_4_Pm	119.51	82.76	201.72	0.218	1.444	14.23
Al_8_Fe_4_Sm	119.99	83.12	202.59	0.219	1.444	14.28
Al_8_Fe_4_Eu	120.49	83.61	203.70	0.218	1.441	14.37
Al_8_Fe_4_Gd	120.63	83.96	204.44	0.218	1.437	14.48
Al_8_Fe_4_Tb	121.16	84.28	205.26	0.218	1.438	14.50
Al_8_Fe_4_Dy	121.41	84.65	206.05	0.217	1.434	14.69
Al_8_Fe_4_Ho	121.83	84.94	206.78	0.217	1.434	14.63
Al_8_Fe_4_Er	122.25	85.24	207.50	0.217	1.434	14.67
Al_8_Fe_4_Tm	122.78	85.25	207.68	0.218	1.440	14.58
Al_8_Fe_4_Yb	112.76	77.80	189.76	0.220	1.449	13.55
Al_8_Fe_4_Lu	117.26	81.02	197.57	0.219	1.447	13.97

## Supplementary Materials

The following are available online at https://www.mdpi.com/1996-1944/12/5/701/s1, Figure S1: The total and partial density of state of Al_8_Fe_4_RE. (**a**) Al_8_Fe_4_Sc; (**b**) Al_8_Fe_4_Y; (**c**) Al_8_Fe_4_La; (**d**) Al_8_Fe_4_Ce; (**e**) Al_8_Fe_4_Pr; (**f**) Al_8_Fe_4_Nd; (**g**) Al_8_Fe_4_Pm; (**h**) Al_8_Fe_4_Sm; (**i**) Al_8_Fe_4_Eu; (**j**) Al_8_Fe_4_Gd; (**k**) Al_8_Fe_4_Tb; (**l**) Al_8_Fe_4_Dy; (**m**) Al_8_Fe_4_Ho; (**n**) Al_8_Fe_4_Er; (**o**) Al_8_Fe_4_Tm; (**p**) Al_8_Fe_4_Yb; (**q**) Al_8_Fe_4_Lu. Figure S2: The Charge density map and differential charge density map of Al_8_Fe_4_RE; (**a**) Al_8_Fe_4_Sc; (**b**) Al_8_Fe_4_Y; (**c**) Al_8_Fe_4_La; (**d**) Al_8_Fe_4_Ce; (**e**) Al_8_Fe_4_Pr; (**f**) Al_8_Fe_4_Nd; (**g**) Al_8_Fe_4_Pm; (**h**) Al_8_Fe_4_Sm; (**i**) Al_8_Fe_4_Eu; (**j**) Al_8_Fe_4_Gd; (**k**) Al_8_Fe_4_Tb; (**l**) Al_8_Fe_4_Dy; (**m**) Al_8_Fe_4_Ho; (**n**) Al_8_Fe_4_Er; (**o**) Al_8_Fe_4_Tm; (**p**) Al_8_Fe_4_Yb; (**q**) Al_8_Fe_4_Lu.
