# Supplementary material for: Phase Stability and Mechanical Properties of Al8Fe4RE via First-Principle Calculations
Source: Materials (Basel). 2019 Feb 27;12(5):701. doi: 10.3390/ma12050701 (PMC6427356; doi:10.3390/ma12050701)
Supplement: Supplementary file 1 [file materials-12-00701-s001.pdf]

materials

MDPI

Article

Phase Stability and Mechanical Properties of Al_8Fe_4RE via First-Principle Calculations

Rongcheng Wang [1,2], Xiaoma Tao [2], Hongmei Chen [2] and Yifang Ouyang [1,2,*]

[1] School of Materials Science and Engineering, South China University of Technology, Guangzhou 510640, China; rong_phy@163.com

[2] Guangxi Key Laboratory of Processing for Non-ferrous Metallic and Featured Materials, School of Physical Science and Technology, Guangxi University, Nanning 530004, China; taoxiaoma@gxu.edu.cn (X.T.); chenhm@gxu.edu.cn (H.C.)

* Correspondence: ouyangyf@gxu.edu.cn

Supplementary data

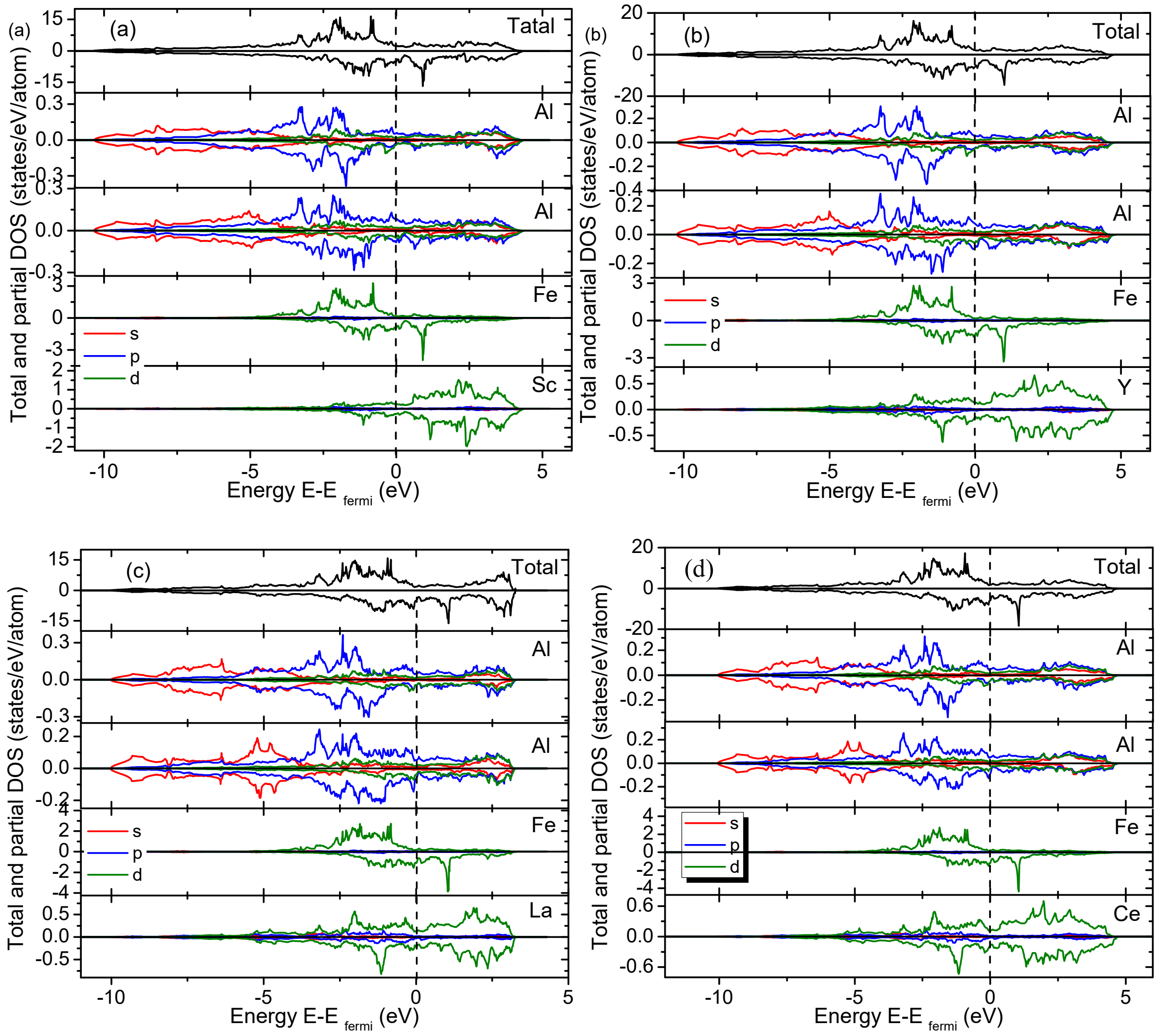

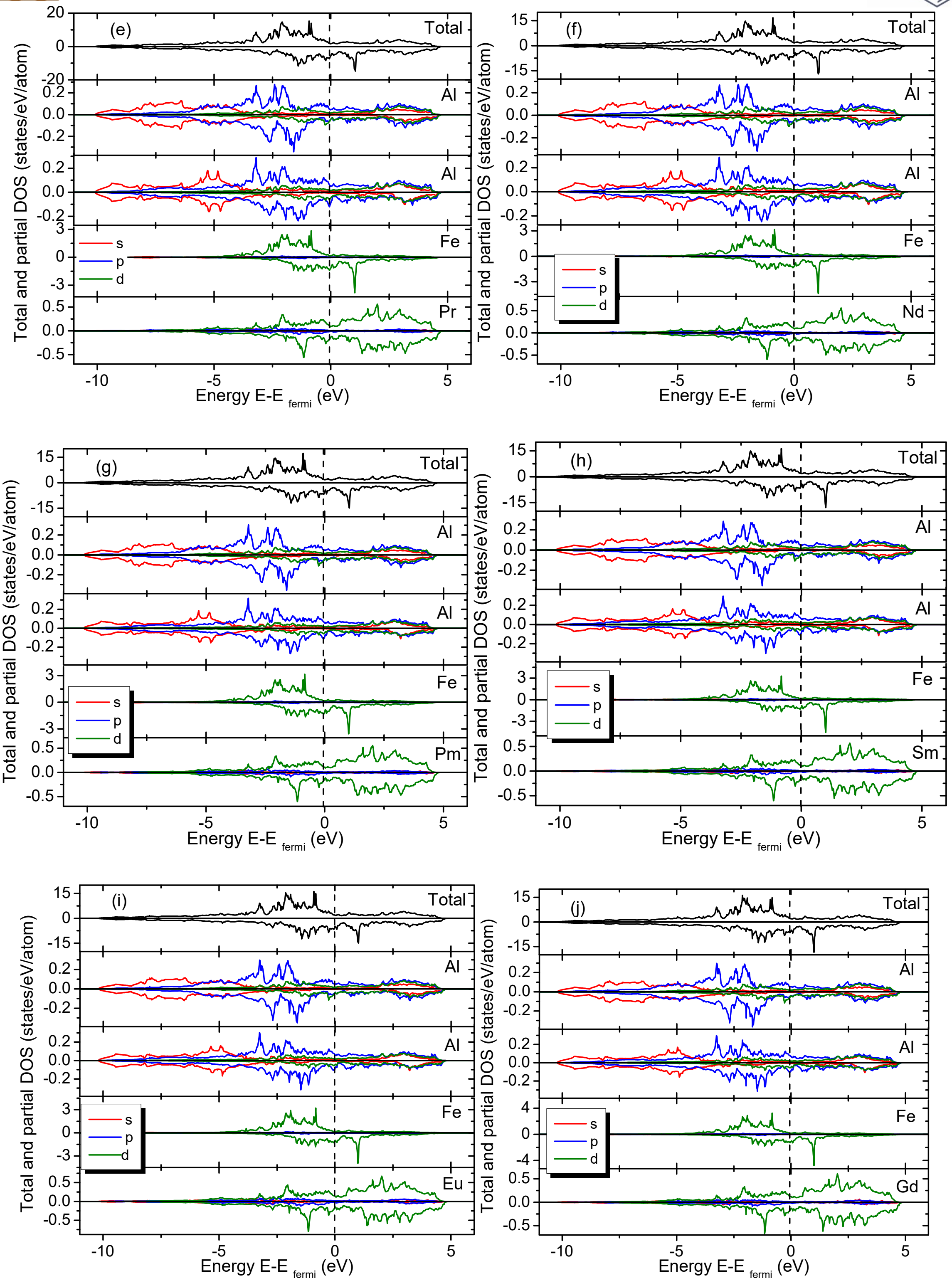
(e)
(f)
(g)
(h)
(i)
(j)
Total
Al
Al
Fe
Pr
Nd
Pm
Sm
Eu
Gd
s
p
d
Total and partial DOS (states/eV/atom)
Energy E-E fermi (eV)

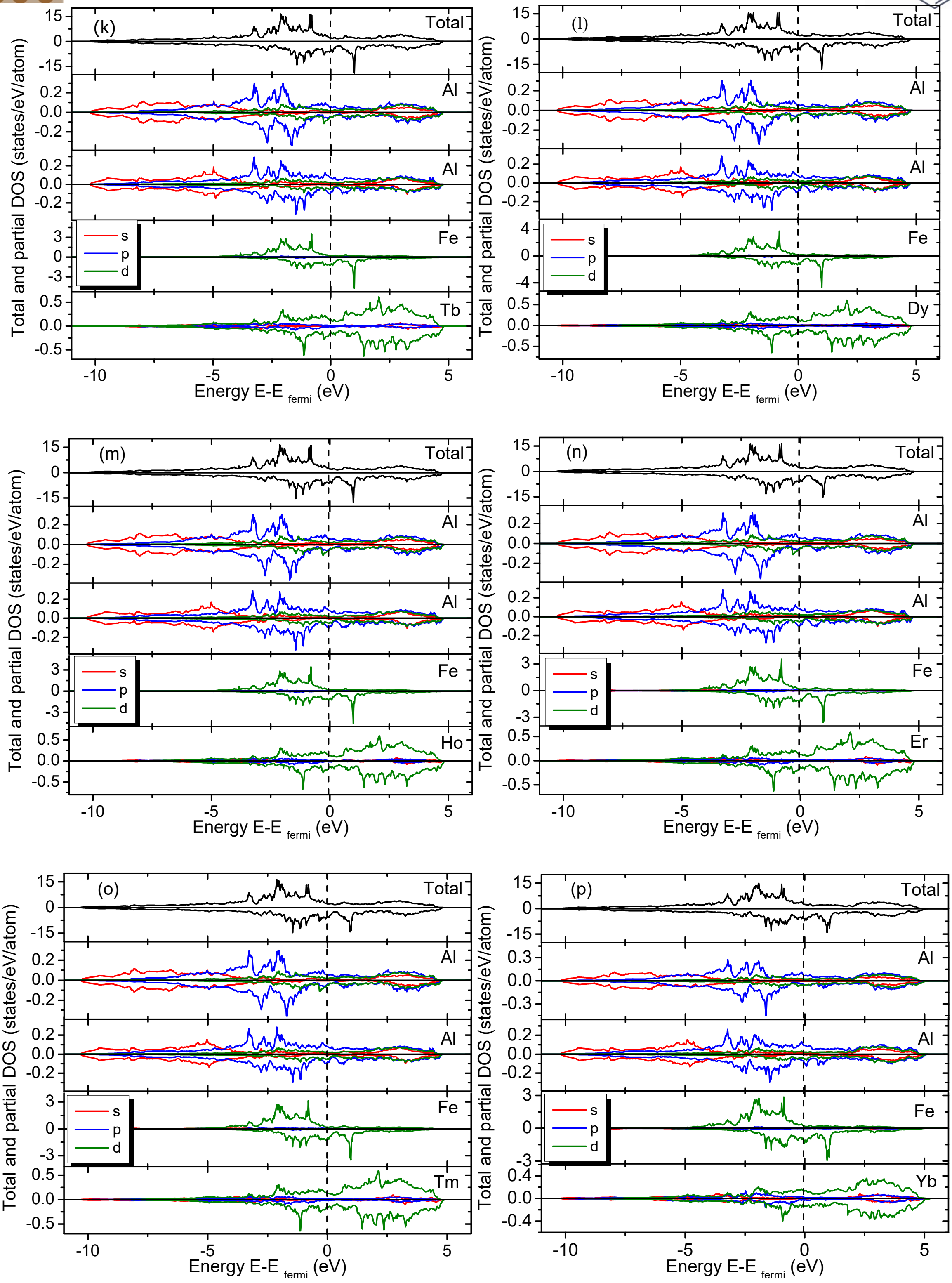

(k)
(l)
(m)
(n)
(o)
(p)
Total
Al
Fe
Tb
Dy
Ho
Er
Tm
Yb
s
p
d
Total and partial DOS (states/eV/atom)
Energy E-E fermi (eV)
15
0
-15
0.2
0.0
-0.2
0.3
-0.3
4
3
-3
-4
0.5
-0.5
0.4
-0.4
-10
-5
5

materials

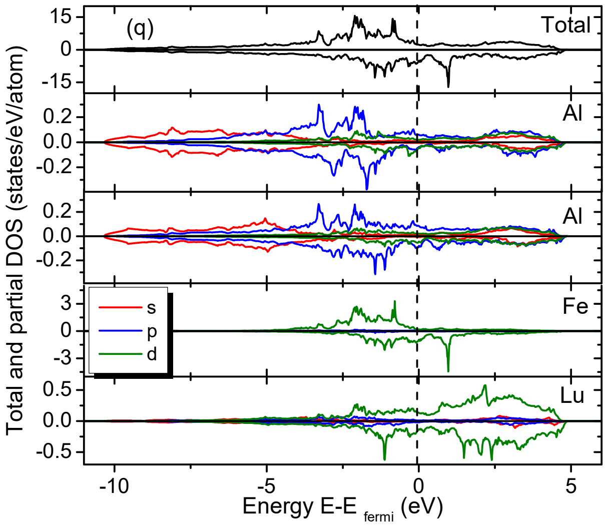

Figure S1. The total and partial density of state of Al_8Fe_4RE. (**a**) Al_8Fe_4Sc; (**b**) Al_8Fe_4Y; (**c**) Al_8Fe_4La; (**d**) Al_8Fe_4Ce; (**e**) Al_8Fe_4Pr; (**f**) Al_8Fe_4Nd; (**g**) Al_8Fe_4Pm; (**h**) Al_8Fe_4Sm; (**i**) Al_8Fe_4Eu; (**j**) Al_8Fe_4Gd; (**k**) Al_8Fe_4Tb; (**l**) Al_8Fe_4Dy; (**m**) Al_8Fe_4Ho; (**n**) Al_8Fe_4Er; (**o**) Al_8Fe_4Tm; (**p**) Al_8Fe_4Yb; (**q**) Al_8Fe_4Lu.

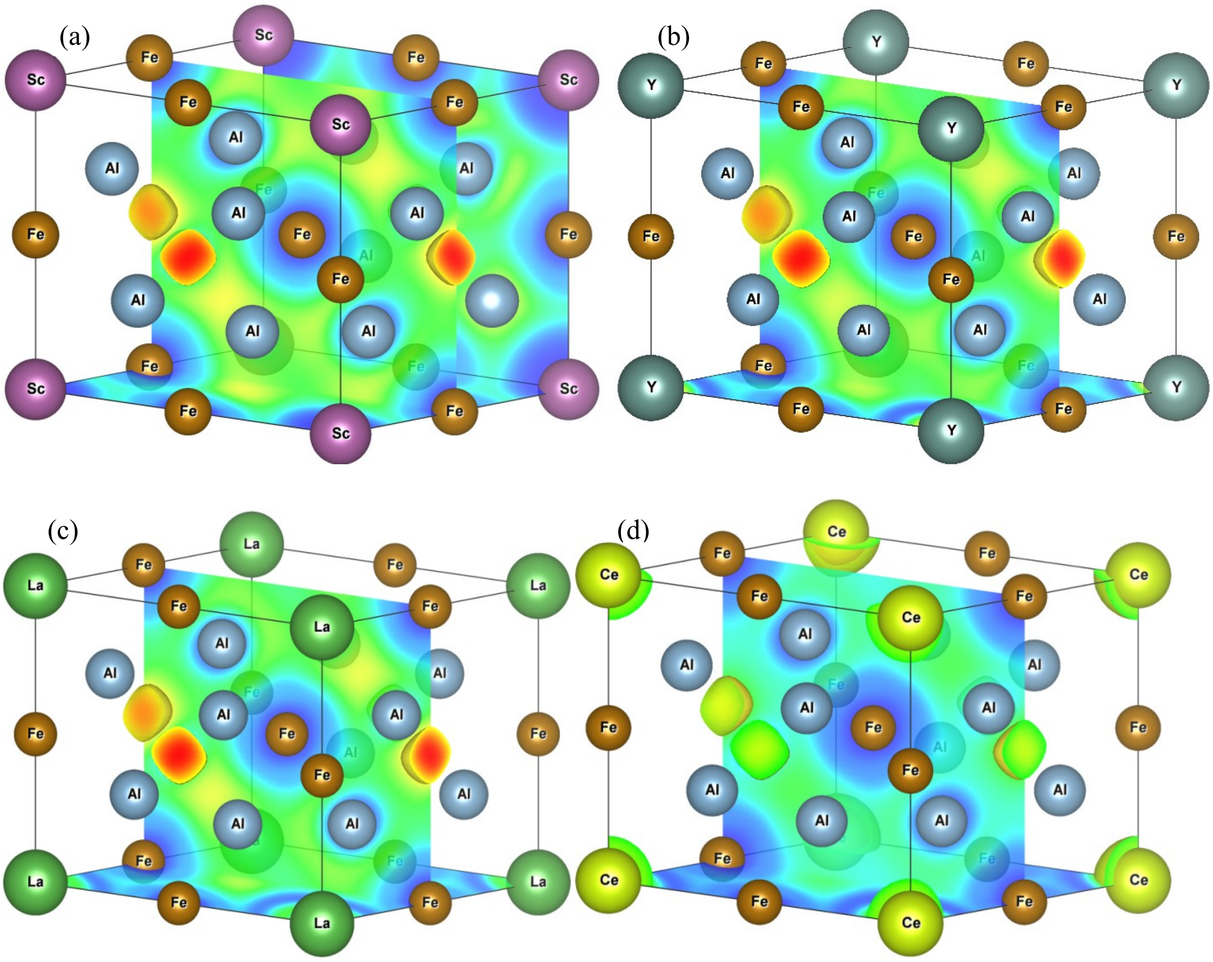

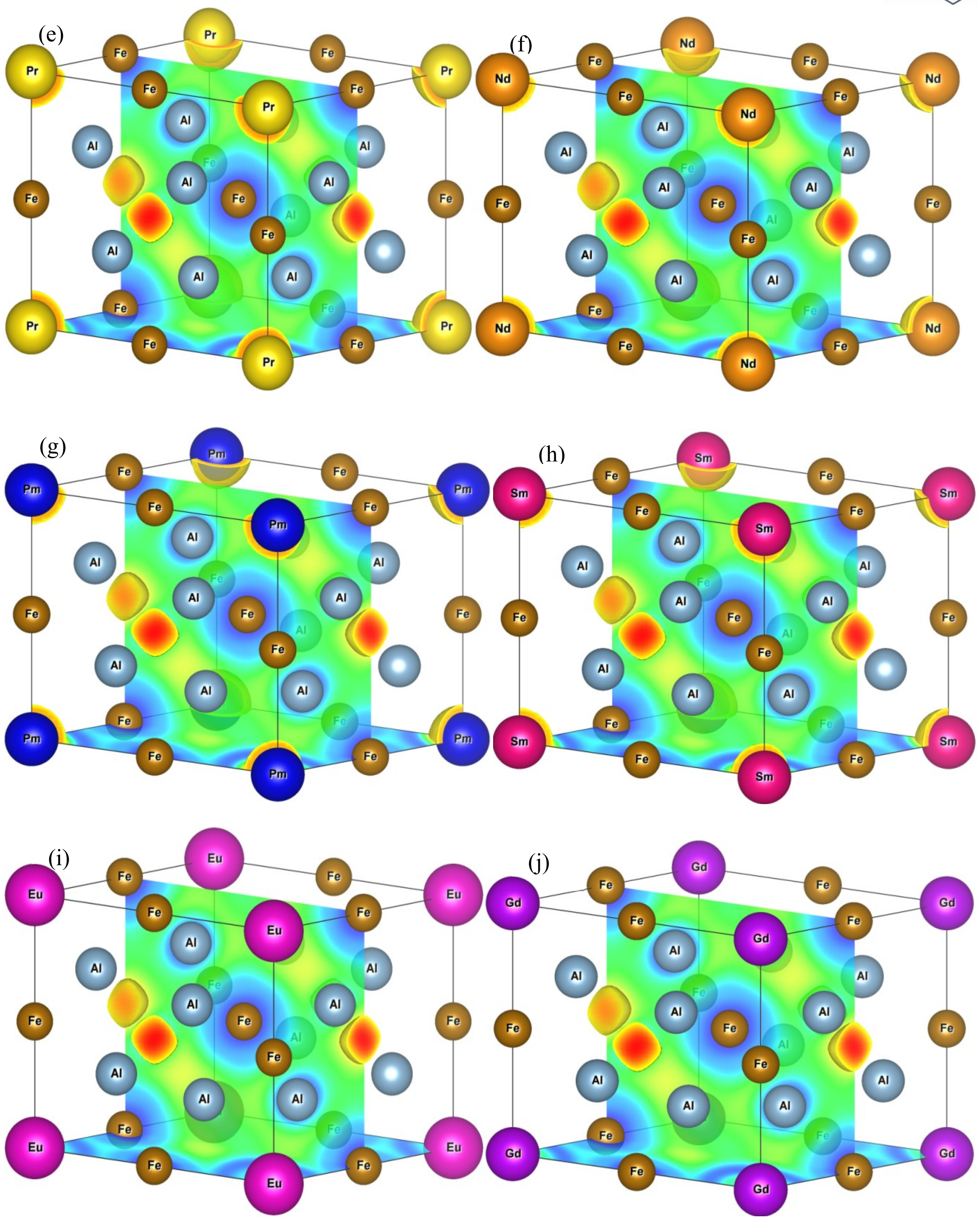
(e)
Pr
Fe
Al
(f)
Nd
Fe
Al
(g)
Pm
Fe
Al
(h)
Sm
Fe
Al
(i)
Eu
Fe
Al
(j)
Gd
Fe
Al

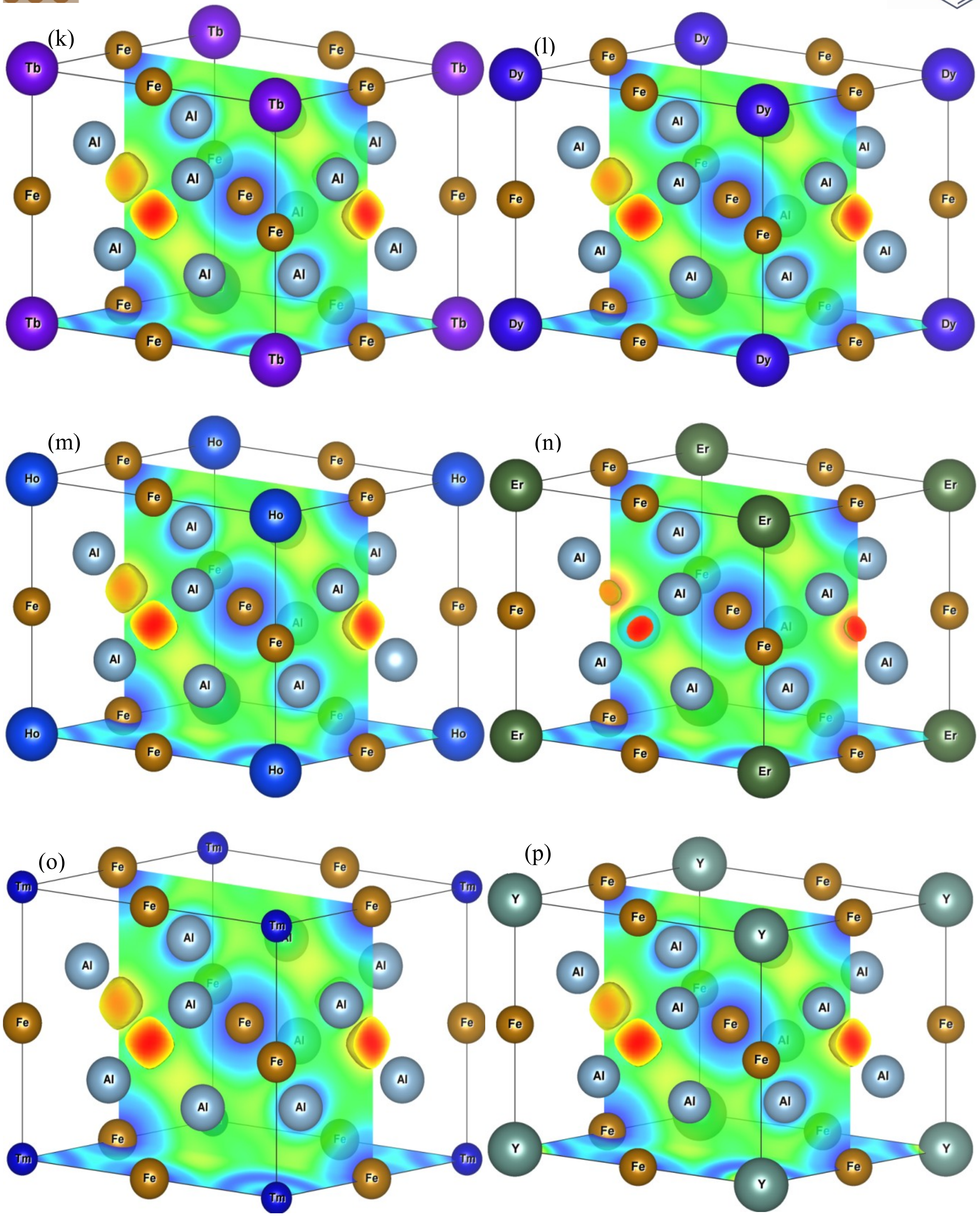
(k)
Tb
Fe
Al
(l)
Dy
Fe
Al
(m)
Ho
Fe
Al
(n)
Er
Fe
Al
(o)
Tm
Fe
Al
(p)
Y
Fe
Al

materials

MDPI

(q)

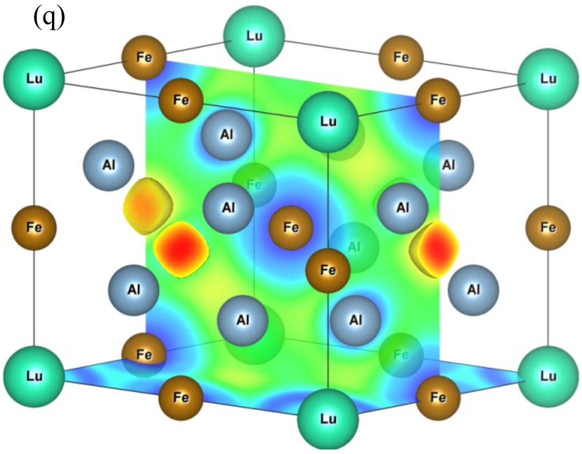

Figure S2. The Charge density map and differential charge density map of Al_8Fe_4RE; (**a**) Al_8Fe_4Sc; (**b**) Al_8Fe_4Y; (**c**) Al_8Fe_4La; (**d**) Al_8Fe_4Ce; (**e**) Al_8Fe_4Pr; (**f**) Al_8Fe_4Nd; (**g**) Al_8Fe_4Pm; (**h**) Al_8Fe_4Sm; (**i**) Al_8Fe_4Eu; (**j**) Al_8Fe_4Gd; (**k**) Al_8Fe_4Tb; (**l**) Al_8Fe_4Dy; (**m**) Al_8Fe_4Ho; (**n**) Al_8Fe_4Er; (**o**) Al_8Fe_4Tm; (**p**) Al_8Fe_4Yb; (**q**) Al_8Fe_4Lu.

© 2019 by the authors. Submitted for possible open access publication under the terms and conditions of the Creative Commons Attribution (CC BY) license (http://creativecommons.org/licenses/by/4.0/).
